# Cooperative p16 and p21 action protects female astrocytes from transformation

**DOI:** 10.1186/s40478-018-0513-5

**Published:** 2018-02-20

**Authors:** Najla Kfoury, Tao Sun, Kwanha Yu, Nathan Rockwell, Kelsey L. Tinkum, Zongtai Qi, Nicole M. Warrington, Peter McDonald, Anuradha Roy, Scott J. Weir, Carrie A. Mohila, Benjamin Deneen, Joshua B. Rubin

**Affiliations:** 1 Department of Pediatrics, Washington University School of Medicine, Campus Box 8208, 660 South Euclid Ave, St Louis, MO 63110 USA; 2 Center for Cell and Gene Therapy, Baylor College of Medicine, Houston, TX USA; 3 Department of Genetics, Washington University School of Medicine, St Louis, MO USA; 4 High Throughput Screening Laboratory, University of Kansas, Lawrence, Kansas, USA; 5 University of Kansas Cancer Center, University of Kansas Medical Center, Kansas City, Kansas, USA; 6 Institute for Advancing Medical Innovation, University of Kansas Medical Center, Kansas City, Kansas, USA; 7 Department of Pathology, Texas Children’s Hospital, Houston, TX USA; 8 Department of Neuroscience, Baylor College of Medicine, Houston, TX USA; 9 Neurological Research Institute at Texas Children’s Hospital, Baylor College of Medicine, Houston, TX USA; 10 Program in Developmental Biology, Baylor College of Medicine, Houston, TX USA; 11 Department of Neuroscience, Washington University School of Medicine, St Louis, USA

**Keywords:** Sex differences, Glioblastoma, Glioma, Rb, p16, p21, Cyclin dependent kinase inhibitors, DNA damage response

## Abstract

Mechanisms underlying sex differences in cancer incidence are not defined but likely involve dimorphism (s) in tumor suppressor function at the cellular and organismal levels. As an example, sexual dimorphism in retinoblastoma protein (Rb) activity was shown to block transformation of female, but not male, murine astrocytes in which neurofibromin and p53 function was abrogated (GBM astrocytes). Correlated sex differences in gene expression in the murine GBM astrocytes were found to be highly concordant with sex differences in gene expression in male and female GBM patients, including in the expression of components of the Rb and p53 pathways. To define the basis of this phenomenon, we examined the functions of the cyclin dependent kinase (CDK) inhibitors, p16, p21 and p27 in murine GBM astrocytes under conditions that promote Rb-dependent growth arrest. We found that upon serum deprivation or etoposide-induced DNA damage, female, but not male GBM astrocytes, respond with increased p16 and p21 activity, and cell cycle arrest. In contrast, male GBM astrocytes continue to proliferate, accumulate chromosomal aberrations, exhibit enhanced clonogenic cell activity and in vivo tumorigenesis; all manifestations of broad sex differences in cell cycle regulation and DNA repair. Differences in tumorigenesis disappeared when female GBM astrocytes are also rendered null for p16 and p21. These data elucidate mechanisms underlying sex differences in cancer incidence and demonstrate sex-specific effects of cytotoxic and targeted therapeutics. This has critical implications for lab and clinical research.

**Electronic supplementary material:**

The online version of this article (10.1186/s40478-018-0513-5) contains supplementary material, which is available to authorized users.

## Introduction

Increasing volumes of data indicate significant sex differences exist in many human diseases [10, 14, 20, 24, 29, 31]. Sex differences in incidence presumably reflect differences in the mechanisms that determine vulnerability to disease [15, 25, 33, 35, 37]. The most recent epidemiological data indicate that cancer occurs at significantly greater frequency in males compared to females [27, 28, 44]. With few exceptions, most notably those cancers that are sex hormone responsive, greater male prevalence is true regardless of age, cancer type, race, or region of the world. Defining the molecular, cellular and organismal bases for sex differences in individual diseases and cancers will determine what, if any, measures need to be taken to tailor precision medicine approaches in a sex-specific manner in order to optimize outcome.

Multiple factors can contribute to sex differences in human health and disease. Significant sex differences in metabolism, gene expression and growth regulation are evident from the moment of fertilization and persist throughout life [36]. From conception on, sex differences arise through the multiple mechanisms of sexual differentiation. Starting with differences in sex chromosome complement and sex specific reprogramming of imprinted loci, and continuing with the organizational or epigenetic effects of in utero and pubertal sex hormones, and finally maturing with the acute action of circulating sex hormones, sex differences are powerfully ensconced at the cellular and organismal levels.

Glioblastoma, the most common malignant brain tumor, occurs with a male to female incidence ratio of 1.6:1 in the United States [27]. While multiple mechanisms may contribute to the incidence difference, prior studies indicate that cell intrinsic sex differences in the response to tumor suppressor loss within the astrocyte lineage may be among the reasons [37]. In these studies, sex differences in regulation of Rb were correlated with differences in clonogenic cell function, proliferation and in vivo tumorigenesis. Here, we investigate how the cyclin dependent kinase (CDK) inhibitors p16, p21 and p27 contribute to sex differences in Rb regulation, tumorigenesis and response to DNA damage. The results of these studies provide new evidence of the importance of considering the biology of sex differences in cancer and more broadly, in any disease with sex differences in incidence or outcome. They advance our understanding of the molecular basis for sex differences in growth regulation and how these differences might underlie the sex differences in cancer incidence. Finally, they suggest that clinical trial design for novel cancer therapeutics should incorporate the possibility of sex differences in response.

## Results

**Fig. 1:**
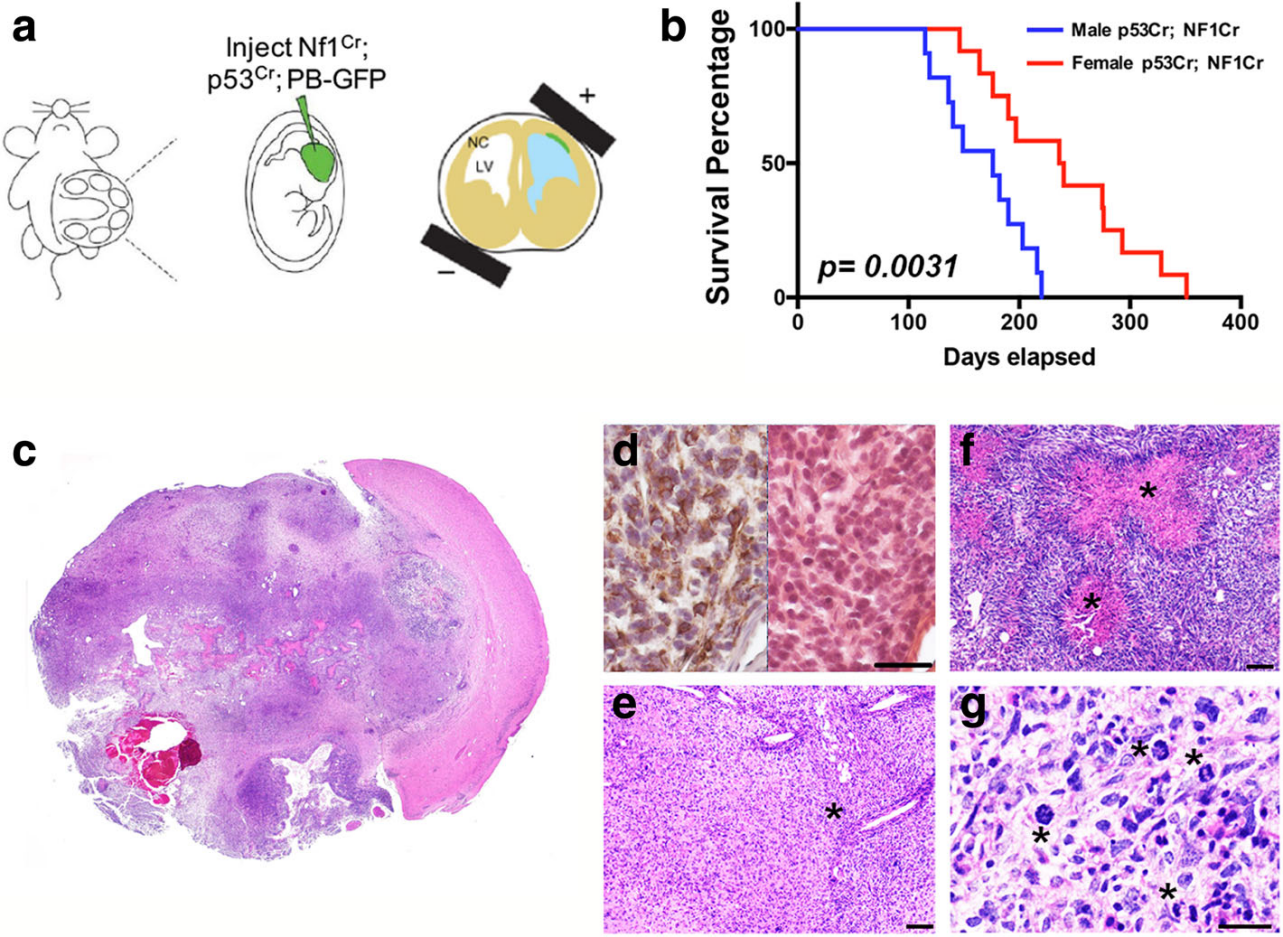
Deletion of neurofibromin and p53 in vivo results in sexually dimorphic gliomagenesis. **a** Schematic of bioelectroporation strategy. The uterus is delivered intact to the external environment (left hand panel). The cerebral ventricles are identified in each pup by trans-illumination and injected with gRNAs directed against neurofibromin and p53 (middle panel). An electric field is imposed across the head of each pup to drive the gRNAs into the sub-ventricular region (right hand panel). **b** Survival was significantly shorter for male mice compared to female mice. While all mice succumbed to tumors, median survival for male mice (*n* = 11) was 176 days and for female mice (*n* = 14), 238 days. *p* = 0.0031 as determined by log-rank test of Kaplan-Meier survival curves. **c** Large invasive tumors formed in all mice. **d** Tumors were diagnosed as astrocytomas based on their expression of glial fibrillary acidic protein (GFAP, brown-right). Corresponding H&E (right) and GFAP (left) staining from a CRISPR IUE brain/tumor section are shown. **e** Tumors were invasive (asterisk) and had other characteristic glioblastoma features like necrosis (**f**), asterisk) and abundant mitoses (**g**), asterisks) evident on examination of hematoxylin and eosin (H&E) stained sections. Scale bars (**e**, **f**) = 100 μM. Scale bar (**d**, **g**) = 50 μM

In published studies, we found that combined loss of neurofibromin and p53 function was transforming for male, but not female astrocytes [37]. To be certain that these sex differences in transformation were not limited to specific features of our model, we sought to determine whether we would see similar results in a different model. We therefore measured in vivo tumorigenesis in male and female Cas9-expressing CD-1 IGS mice following in utero electroporation of gRNAs targeting the *Nf1* and *p53* genes into peri-ventricular neural progenitors. Two pX330 vectors [13] with guide sequence inserts targeting *Nf1* and *p53* were injected into the lateral ventricles of embryonic day 15–16 pups (E15–16), and progenitor cells were targeted via bioelectroporation. This model is referred to as the CRISPR-IUE Glioma Model [21] (Fig. 1a and Additional file 1: Figure S1A). All male and female mice receiving the CRISPR IUEs were euthanized for neurological deficits or excessive weight loss. In all cases, large tumors were confirmed by direct inspection and histopathology. While combined loss of neurofibromin and p53 function was tumorigenic in 100% of male and female mice, the process was accelerated in male mice in which median survival was 176 days compared to 238 days for female mice (Fig. 1b). The sex differences in survival were statistically significant with a *p*-value of 0.0031 as determined by Log-Rank test. Both male and female tumors were characteristically large at the time of euthanasia (Fig. 1c) and diagnosed as glioblastoma-like based on expression of glial fibrillary acidic protein (Fig. 1d), and standard histological features including invasion (Fig. 1e), necrosis (Fig. 1f) and abundant mitoses (Fig. 1g). In general, female tumors exhibited more necrosis than male tumors, while male tumors exhibited more rosettes, a primitive neuro-ectodermal (PNET)-like features (Additional file 1: Figure S1B). Increased necrosis has also been reported to be a more prominent feature in female patient pathological specimens and magnetic resonance imaging [9, 12]. Together, these data indicate that sex differences in tumorigenesis after combined loss of neurofibromin and p53 function are evident in different mouse strains and regardless of whether the loss is engineered in vitro or in vivo and independent of the method by which p53 function is abrogated.

**Fig. 2:**
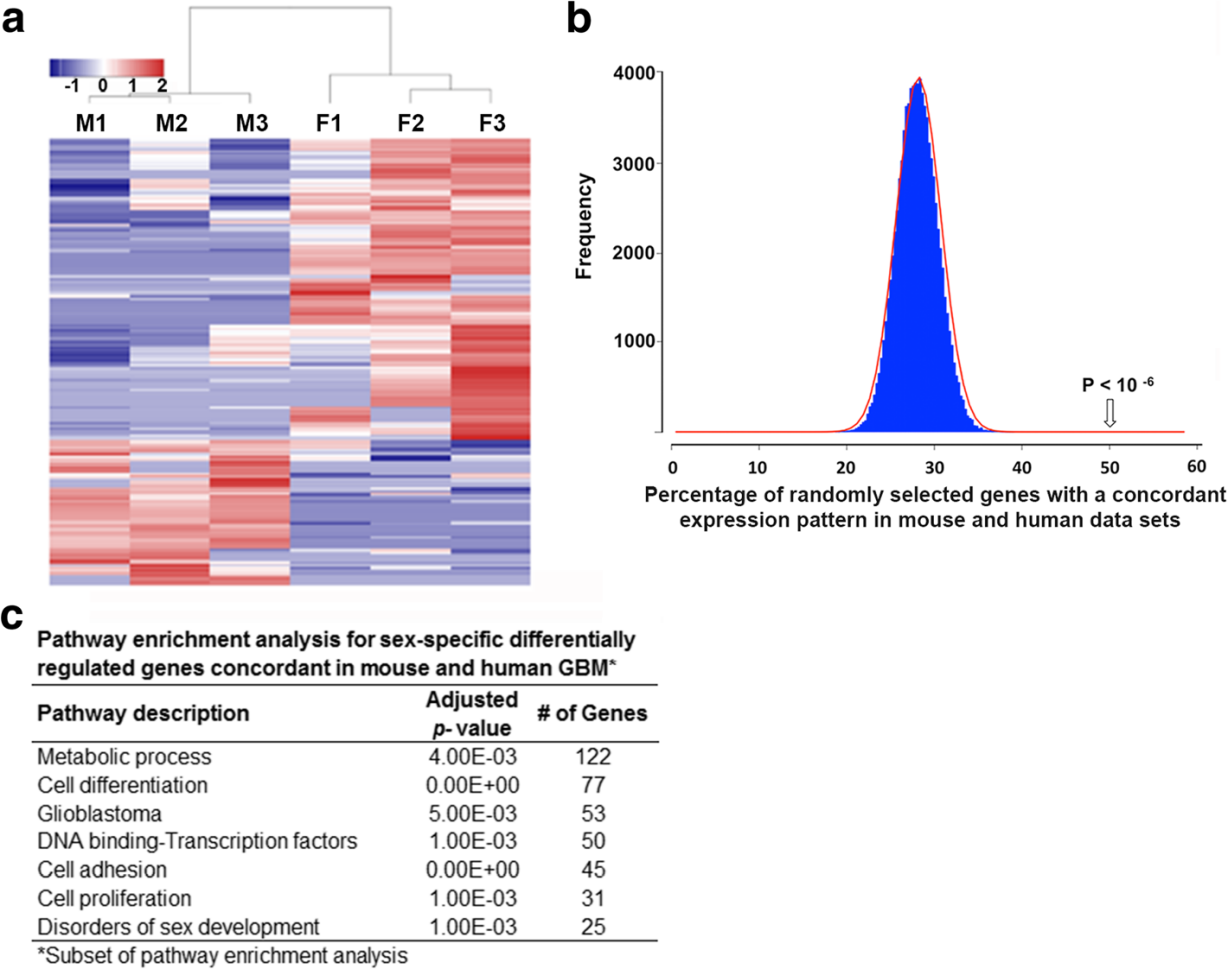
Male and female GBM astrocytes exhibit transcriptome-wide differences. **a** Heatmap of male and female differentially regulated genes with 2-fold or greater change in expression. **b** Histogram plot depicting a probability of 10^− 6^ for a concordance of 50% in gene expression patterns in mouse and human GBM data sets. **c** Pathway analysis of differentially regulated genes with concordant expression patterns between mouse and human GBM was performed using Genomatix GePS

To gain further insights into the molecular basis for these differences in male and female cell behaviors, we performed transcriptomic analyses using RNA sequencing in astrocytes rendered null for neurofibromin and p53 function. Briefly, male and female astrocytes were isolated from the neocortices of postnatal day *1 Nf1fl/fl GFAP-Cre* mice and genotyped for sex using Jarid1c and Jarid1d PCR. Male and female *Nf1−/−* astrocytes were then infected with retrovirus encoding a flag-tagged dominant-negative form of p53 (DNp53) and EGFP resulting in male and female astrocytes null for Nf1 and p53 function [37]. The DNp53 plasmid consists of amino acids 1–14 of the transactivation domain followed by amino acids 303–393 thus lacking the DNA binding domain. These astrocytes serve as a model of GBM and we refer to them as GBM astrocytes. We obtained high quality RNA sequencing data as characterized by number of input reads (3.4 to 4.1 × 10^6^/sample) and the number of uniquely mapped reads (74–84%). Out of 2567 differentially regulated genes between male and female GBM astrocytes (Female/Male *Nf1−/−;DNp53*), 594 were statistically significant at FDR < 0.05 (Fig. 2a). Consequently, we wanted to investigate whether these transcriptome-wide sex differences in our murine GBM model are also present in human GBM. To do so, we mined the TCGA GBM data sets and found a concordance in expression differences in 49% of these significantly differentially regulated genes. To determine whether this concordance in expression between mouse and human GBM samples could be due to chance, we randomly selected 100,000 different sets of 500 mouse genes, and measured the percent of genes that exhibited concordant sex-specific gene expression. We found that on average approximately 28% of genes exhibit concordance by chance. We next calculated the probability of observing a 49% concordance, and found it to be 10^− 6^. We therefore concluded that the observed sex differences in gene expression in our murine GBM model are representative of sex differences in gene expression that are present in human GBM (Fig. 2b). Pathway enrichment analysis for the concordant differentially regulated genes was performed using a combination of KEGG pathway and Genomatix Pathway System (GePS). Relevant and important sexually dimorphic pathways identified, included cell differentiation, cell adhesion, glioblastoma, proliferation, disorders of sex development, and DNA-binding transcription factors (Fig. 2c). This is the first demonstration of transcriptome-wide sexual dimorphism in a cancer model and it suggests that a great breadth of differences between male and female cells could contribute to differences in their susceptibility to malignant transformation.

**Fig. 3:**
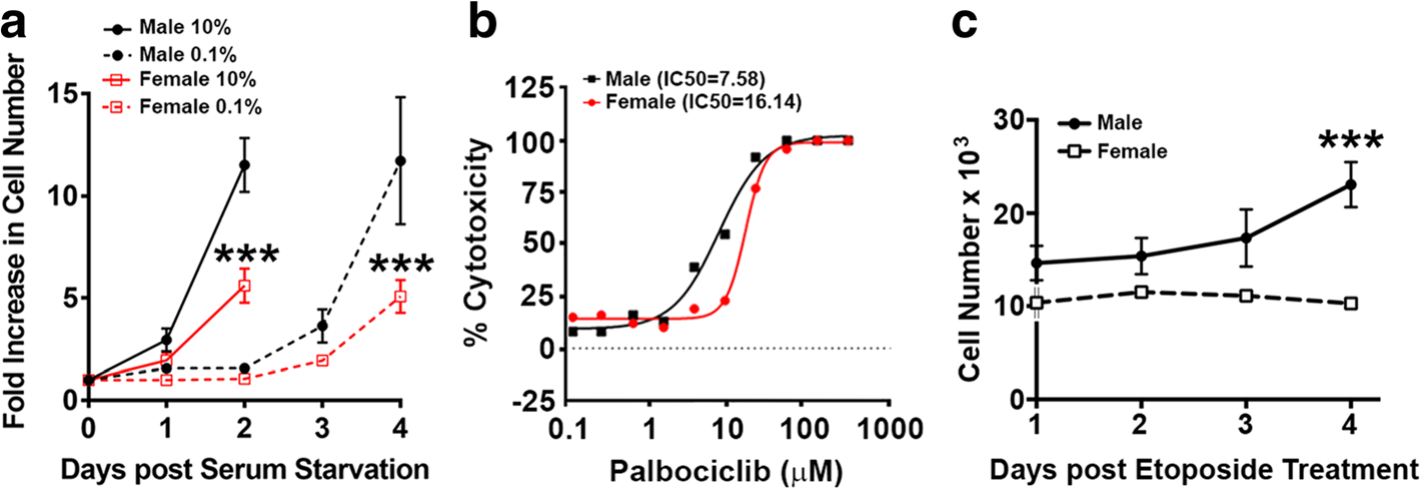
Male and female GBM astrocytes display sex differences in response to serum deprivation, CDKi treatment and chemotherapy. **a** Male and female GBM astrocytes were grown in either 10% or 0.1% serum and cell number was measured over the course of 96 h by trypan blue exclusion. The differences in growth rates (*n* = 3) in 0.1% serum was significant with *p* < 0.0001 as determined by two-way ANOVA. **b** Representative dose response curve for palbociclid treatment of male and female GBM astrocytes. Cell viability was measured with CellTiter Glo. IC50 values were calculated using four-parameter non-linear regression analysis in Graphpad Prism. **c** Male and female GBM astrocytes were treated with etoposide (10 μg/ml) or vehicle for 24 h. At that time, media was changed to 10% FBS supplemented DMEM/F12 and cell number was measured over the course of 4 days by trypan blue exclusion. The differences in growth rates were significant with *p* = 0.0001 as determined by two-way ANOVA (*n* = 3)

In prior work, we determined that sex differences in in vivo tumorigenesis of astrocytes rendered null for neurofibromin and p53 function were the result of sexual dimorphism in Rb function. Upon combined loss of neurofibromin and p53 function, female cells exhibited a compensatory protective effect that was evident in greater inhibition of Rb phosphorylation, lower growth rates and levels of clonogenic cell function, and less in vivo tumorigenesis, compared to their male counterparts. These sex differences disappeared when Rb and p53 function were simultaneously abrogated by transformation with SV40 large T antigen [38]. Thus, to begin an investigation into the mechanisms by which female cells were protected from transformation upon loss of neurofibromin and p53 function, we examined the cell growth rate of astrocytes rendered null for both under basal and experimental conditions (serum deprivation, cyclin dependent kinase inhibitors and chemotherapy). Consistent with our prior findings [37], under 10% serum containing conditions, male GBM astrocytes proliferate more than their female counterparts (Fig. 3a). Upon serum deprivation (0.1%), female GBM astrocytes undergo almost complete growth arrest while male GBM astrocytes continue to increase in cell number, albeit at a greatly diminished rate. Next, we asked whether we could detect sex differences in cyclin dependent kinase inhibitor (CDKi) cytotoxicity such as PD0332991 (Palbociclib). To measure cytotoxicity, we counted cell number following Palbociclib treatment using cell TiterGlo assay. Palbociclib was more effective in male GBM astrocytes (Fig. 3b). Finally, to determine the effect of DNA damage on growth in culture, we treated GBM astrocytes with etoposide. Etoposide treatment resulted in growth arrest in female, but not male GBM astrocytes (Fig. 3c). Altogether, these data reveal that male and female GBM cells respond differently to a variety of conditions that would be expected to inhibit Rb phosphorylation and enhance its function.

**Fig. 4:**
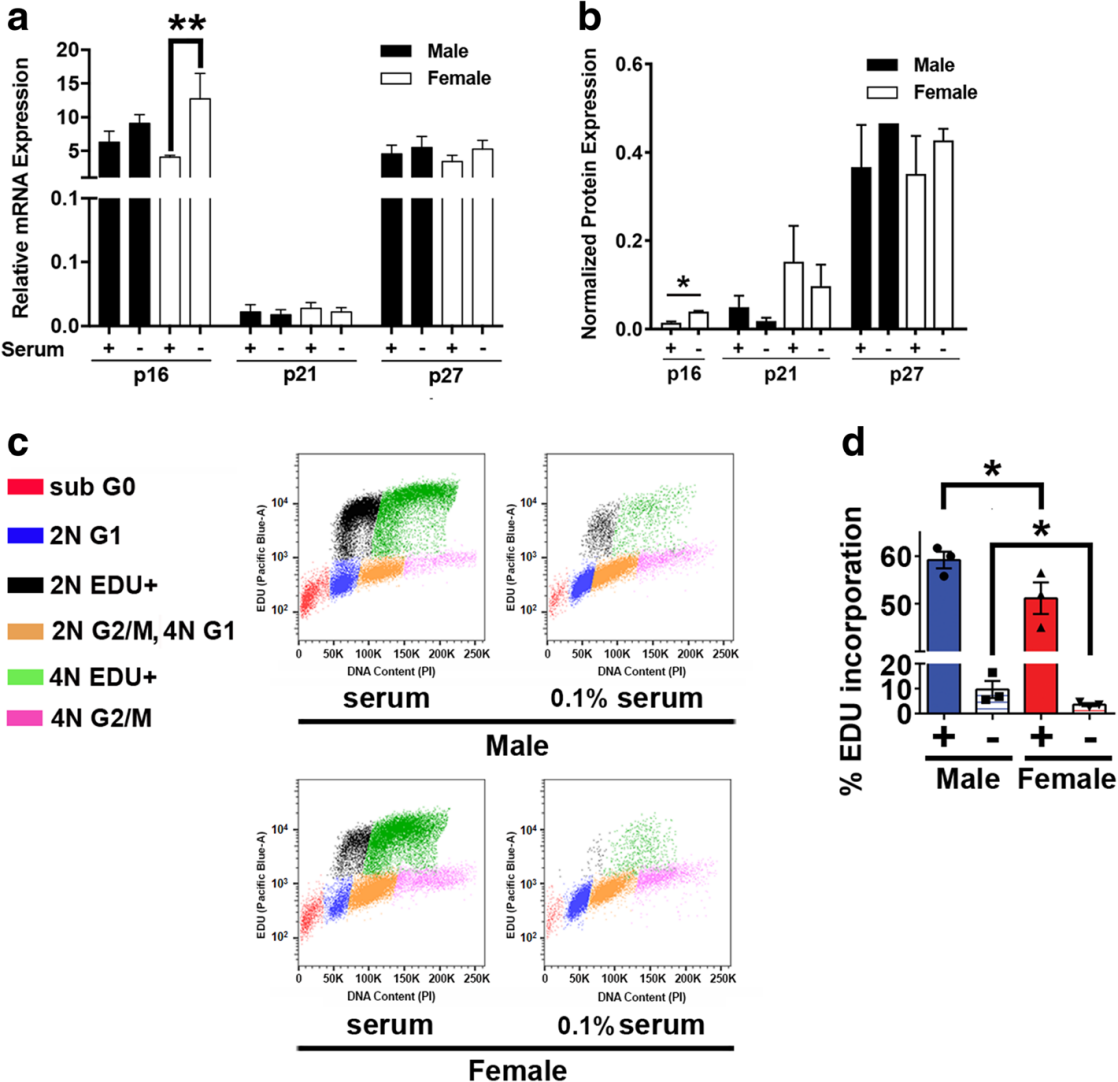
Female GBM astrocytes exhibit greater capacity for p16 induction and growth inhibition in response to serum starvation. **a** p16, p21 and p27 expression was measured by quantitative PCR in male and female GBM astrocytes grown in the presence or absence of 10% serum. Female GBM astrocytes express higher levels of p16 in response to serum withdrawal (*n* = 3 independent litters, *p* < 0.05 as determined by one-way ANOVA and post-hoc Dunnett’s test). **b** Expression of p16, p21 and p27 protein was measured by Western blot analysis in the presence or absence of 10% serum (+). Means and SEM of protein expression was calculated from three independent experiments. Values were normalized within each experiment to male control. ** = *p* < 0.005 as determined by one-way ANOVA with Sidaks’ multiple comparisons test. **c** Flow cytometric analysis of cell cycle distribution of EDU-labelled male and female GBM astrocytes indicated that male and female cells contain 2 N and 4 N sub-populations and that under 10% serum containing conditions both are synthesizing DNA. Upon serum deprivation (0.1%), male, but not female, GBM astrocytes continue to incorporate EDU into both the 2 N and 4 N populations albeit at substantially lower levels than control. **d** Under basal serum containing conditions, male GBM astrocytes incorporate significantly greater levels of EDU than their female counterparts (*n* = 3,* = *p* = 0.01 as determined by paired *t*-test). Upon serum deprivation, male cells incorporate significantly more EDU than female cells (* = *p* = 0.03 as determined by paired *t*-test)

To investigate the mechanism underlying these sexually dimorphic responses, we first examined the expression of regulators of Rb (p16, p21 and p27) under these same conditions. When cultured in serum containing media, there was equivalent expression of p16 mRNA in both male and female GBM astrocytes (Fig 4a), but little to no p16 protein expression in either (Fig. 4b). While abrogation of p53 function results in a dramatic reduction in p21 expression, small levels of p21 expression remain detectable in both male and female GBM astrocytes. Under basal serum containing conditions, female cells exhibited consistently higher levels of p21 protein expression compared to male GBM astrocytes (Fig. 4b), and equivalent levels of p27 mRNA and protein expression (Fig. 4a and b). To further evaluate sex differences in p16, p21 and p27 expression, we performed Western blot analysis of male (*n* = 4) and female (*n* = 4) in vivo GBM tumor tissue recovered from the CRISPR-IUE glioma model. While p16 protein was undetectable in these samples (data not shown), female tumors expressed significantly greater levels of p21 and trended towards greater expression of p27 (Additional file 1: Figure S2), thus, confirming sex differences in these critical negative regulators of growth. Additionally, concordant gene expression changes with known interactors of p16, p21 and p53 were also identified. These include sonic hedgehog (Shh) and Cyclin D1 (p16 interactors), p63 (p21 interactor), and BTG Anti-Proliferation Factor 2 (Btg2) and High Mobility Group AT-Hook 2 (Hmga2) (p53 interactors). Consistent with the in vivo tumorigenesis data, tumor suppressor genes such as Btg2 and p63 were more highly expressed in females GBM astrocytes compared to their male counterparts, while genes involved in tumor progression and invasion such as Hmga2 and shh were more highly expressed in male GBM astrocytes compared to their female counterparts.

p16, p21 and p27 mediate Rb regulation in response to stressors like changes in growth factor availability and DNA damage [5, 7]. To test whether there were differences in the responsiveness of these regulators to these stressors, we examined the expression of p16, p21 and p27 after serum withdrawal or induction of DNA damage with etoposide. Upon withdrawal of serum for 48 h, p16 mRNA and protein levels were significantly elevated in female, but not male GBM astrocytes (Fig. 4a, b). Serum deprivation resulted in a decline in p21 protein expression in both male and female GBM astrocytes and was without effect on p27 expression (Fig. 4a, b). We further evaluated proliferation in 10% and 0.1% serum containing conditions with cell cycle analysis and EdU incorporation. Under 10% serum containing conditions, both male and female GBM astrocytes are comprised of 2 N and 4 N sub-populations, each of which incorporated EDU at a high, but significantly different rates (Male > Female (Fig. 4c, d)). Upon serum deprivation (0.1%), little EDU incorporation is evident in the female GBM astrocytes while substantially greater incorporation of EDU is evident in both the male GBM astrocyte 2 N and 4 N populations (Fig. 4c, d).

**Fig. 5:**
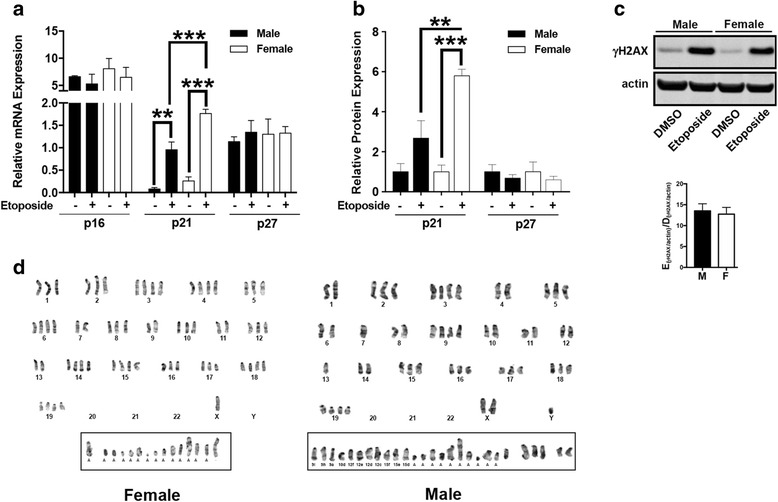
Female GBM astrocytes exhibit greater capacity for p21 induction and growth inhibition in response to etoposide. **a** The effect of etoposide treatment (10 μg/ml for 24 h) on p16, p21 and p27 RNA expression was measured by quantitative PCR. Etoposide induced p21 mRNA expression in both male and female GBM astrocytes but the level of increase was greater in females compared to male GBM astrocytes. **b** Expression of p16, p21 and p27 protein was measured by Western blot analysis. Means and SEM of protein expression was calculated from three independent experiments. Values were normalized within each experiment to male control. ** = *p* < 0.005 and *** = *p* < 0.0005 as determined by one-way ANOVA with Sidaks’ multiple comparisons test. **c** Etoposide treatment resulted in equivalent induction of histone H2AX phosphorylation (γH2AX) in male and female GBM astrocytes. Shown is a representative Western blot and accompanying quantification of three independent experiments. **d** Etoposide had greater clastogenic effects in male compared to female GBM astrocytes. Shown are representative metaphase spreads of etoposide-treated male and female GBM astrocytes. Male and female GBM astrocytes contained 2 N and 4 N subpopulations. Etoposide induced chromosomal fragmentation in both sexes but there was a substantially greater clastogenic effect in male compared to female GBM astrocytes

To determine the effect of DNA damage on p16, p21 and p27 expression, we treated GBM astrocytes with etoposide. In response to etoposide there was a substantial increase in p21 mRNA (Fig. 5a) and protein (Fig. 5b) levels in both male and female GBM astrocytes, but the increase was greater and more significant in female, compared to male cells. p27 protein was highly expressed in GBM astrocytes and etoposide treatment reduced its expression in both male and female cells. There was no detectable p16 protein expression under these basal or etoposide treated conditions (Fig. 5a and b).

To determine whether etoposide treatment was inducing equivalent amounts of DNA damage, we examined levels of γH2AX, a biomarker of DNA double strand breaks, by Western blot after etoposide treatment. We found that there were equivalent levels of γH2AX, suggesting that the differences in response to etoposide were not a consequence of differences in the initial levels of DNA double strand breaks (Fig. 5c). Etoposide is a well-known clastogen [23] and to determine whether there were sex differences in etoposide-induced chromosomal aberrations, we examined metaphase spreads of DMSO and etoposide-treated GBM astrocytes. Karyograms of three metaphase spreads of each of three male and female GBM astrocytes preparations treated with etoposide or DMSO were examined in detail for aneuploidy and structural changes. Consistent with the aneuploidy observed in the cell cycle analysis, under DMSO control conditions, both male and female GBM astrocytes contained subpopulations with chromosome numbers that varied between 50 and 120, and 41 and 111 respectively (Additional file 1: Figure S3). Little to no chromosomal structural variants were observed in either male or female GBM astrocytes treated with DMSO. In contrast, etoposide treatment resulted in the acquisition of chromosomal anomalies in both male and female GBM astrocytes (Fig. 5d). In males, the numbers of fragments ranged from 19 to 30 and in females it ranged between 6 and 15. This difference reached statistical significance (*p* = 0.02, two-tailed t-test-value for difference). Taken together, these data suggest that female GBM astrocytes might be protected from transformation through the maintenance of more normal cell cycle regulation in response to growth factor availability and genotoxic stress. Further, these data suggest that this might be due to maintenance of p16 and p21 function.

**Fig. 6:**
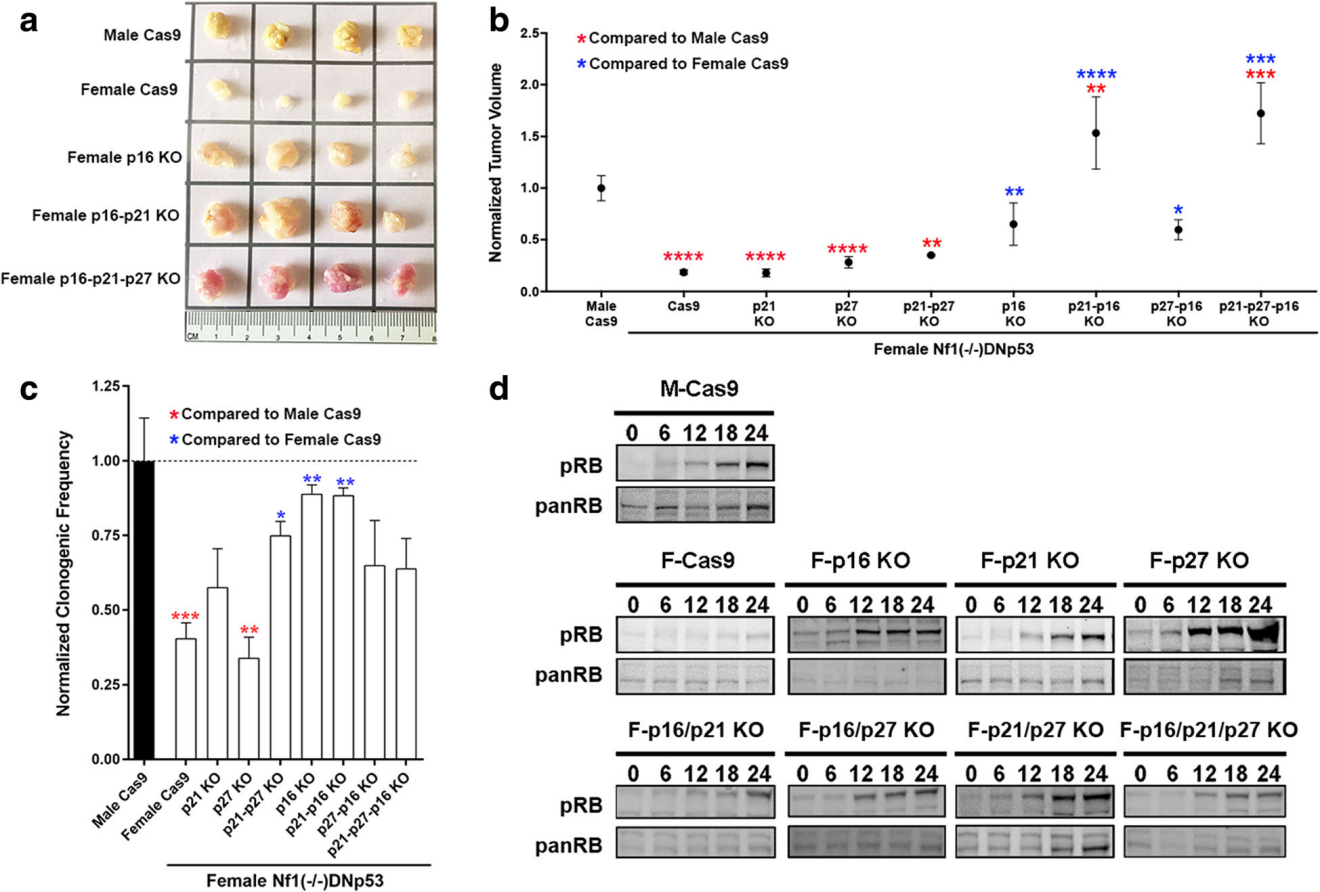
Combined loss of p16 and p21 in female GBM astrocytes recapitulates the male GBM phenotype. **a** Representative flank tumors from male and female GBM Cas9 control astrocyte initiated tumors. **b** Quantification of mean and SEM tumor volumes of male and female GBM Cas9 control tumors and each of the p16, p21 and p27 single and combinatorial KO female cell lines. Tumors were harvested at 8-weeks post-implantation and measurements are of ex vivo tumors. Statistical significance was determined using either male Cas9 tumors as reference (red asterisks) or female Cas9 tumors as reference (blue asterisks). Asterisks (1–4) refer to *p* values of < 0.05, < 0.005, < 0.0005, or < 0.00005 as determined by one-way ANOVA and Dunnett’s post-hoc test (*n* = 15 for *p21 KO* and *p27 KO*, and *n* = 5 for each of *p21;p27 DKO*, *p16 KO*, *p21-p16 DKO*, *p27-p16 DKO* and *p21-p27-p16 TKO*). **c** ELDA assays were utilized to measure clonogenic cell frequency. Asterisks refer to comparisons between male and female Cas9 GBM cells and *p*-values are as described for panel b. **d** The effect of p16, p21 and p27 loss on Rb phosphorylation was measured by Western blot analysis of cells stimulated with serum after 48 h of serum starvation. Shown are representative blots from individual experiments

To assess the independent and potentially combinatorial roles of p16, p21 and p27 in protecting female cells from transformation, we abrogated expression of each alone, and in every combination using CRISPR/Cas9. The effects of each manipulation on transformation were evaluated using in vivo tumorigenesis and two underlying cellular behaviors previously identified as distinguishing male and female functions in this model: clonogenic cell frequency and regulation of Rb phosphorylation. To test in vivo tumorigenesis, we injected 1 million male or female Cas9 control cells or the same number of female Cas9 cells also expressing sgRNAs to p16, p21, or p27 alone or in combinations as indicated, into the flanks of NCR nude mice (*n* = 15 for *p21 KO* and *p27 KO*, and *n* = 5 for each of *p21-p27 DKO*, *p16 KO*, *p21-p16 DKO*, *p27-p16 DKO* and *p21-p27-p16 TKO*). Loss of target gene expression was determined in each case by Western blot analysis (Additional file 1: Figure S4). As we had previously shown and consistent with the IUE model (see Fig. 1), female Cas9 control cells were less competent to form tumors than male Cas9 control cells (Fig. 6a and b)**.** Individual and combined loss of p21 and p27 was without substantial effect on in vivo tumorigenesis. In contrast, p16 loss alone significantly increased female cell tumorigenesis, though not to male levels. However, combined loss of p16 and p21, or combined loss of p16-p21-p27 did render female cells as competent for in vivo tumorigenesis as male cells. Surprisingly, loss of p27 expression reduced the tumorigenic effect of p16 loss and was without effect on the combined p16 and p21 phenotype (Fig. 6b). Together, these data suggested that the relative cell intrinsic protective effect in female astrocytes was likely to be mediated by differences in the activities of p16 and p21.

To further explore the cellular behaviors associated with the differences in in vivo tumorigenesis, we next measured in vitro clonogenic cell frequency using Extreme Limiting Dilution Assay (ELDA). We found that p16 alone, but not p21 or p27 alone, significantly increased clonogenic cell frequency to levels comparable to male GBM astrocytes (Fig. 6c). In contrast to the tumorigenesis results, p27 enhanced the effects of p21 loss on clonogenic cell frequency to near male levels, and did not diminish the effect of p16 deletion. Finally, we examined Rb phosphorylation. As is standard, we measured the time course and magnitude of Rb phosphorylation upon adding serum back after 48 h of starvation in all of the cell lines. We found that deletion of p16 or p21 or p27 alone increased Rb phosphorylation to levels that were comparable to male Cas9 controls (Fig. 6d). Combinatorial loss of p16, p21 and p27 had no additional effect on Rb phosphorylation.

## Discussion and conclusions

The motivation for this study was to understand the mechanisms underlying sex differences in cancer incidence and to explore the implications of these differences on therapeutic responses. Cancer occurs more frequently in males than in females overall [34]. With few exceptions, this is true for all ages, all races, and most tissues and cell types common to males and females. It is particularly evident for tumors of the brain and spinal cord, especially those of neuro-ectodermal origin [27]. Moreover, more males die from cancer than females [34]. At the molecular level, there must be discernible mechanisms by which sexual differentiation interacts with the pathways of tumorigenesis to create the sex bias in cancer rates. The fact that there are significant disparities in the rates and outcomes of cancers between prepubertal boys and girls [22, 26, 35] indicates that the biology of sex differences as it relates to cancer risk cannot be limited to the acute actions of sex hormones, and must also be a consequence of one or both of two additional effectors of sexual differentiation: 1) gene dosage differences as a consequence of incomplete X chromosome inactivation in female cells, and expression from the non-pseudo-autosomal component of the Y chromosome in male cells, and 2) the organizational or epigenetic actions of in utero and perinatal sex hormones in patterning gene expression and phenotype for life [4].

In prior studies, we found that sex differences in a murine model of glioblastoma were the result of sexual dimorphism in Rb regulation that emerged upon combined loss of neurofibromin and p53 function [37]. In the current study, our goal was to define the molecular basis for the differences in Rb regulation and to investigate its relatedness to sex differences in tumorigenesis.

Key new data in this study include the demonstration that sex differences in the tumorigenic effects of combined neurofibromin and p53 loss are evident across mouse strains, independent of how neurofibromin and p53 loss of function is engineered, and insensitive to whether the loss occurs in vivo or in vitro. The resultant male and female GBM astrocytes exhibit significant transcriptome-wide differences in gene expression that mirror gene expression differences in patient specimens. A number of important pathways that warrant evaluation in future studies were identified in this cross-species analysis. In addition, in the absence of p53, female GBM astrocytes exhibit greater genomic stability than their male counterparts. Together, these data provide essential validation of the model for exploring the molecular mechanisms involved in sex differences in tumorigenesis. From these data, we conclude that both p16 and p21 function are essential to protect female GBM astrocytes from transformation upon combined loss of neurofibromin and p53 function. While p16 was the only CDK inhibitor whose loss alone resulted in an increase in the clonogenic cell fraction of female GBM astrocytes to levels that were comparable to male Cas9 control levels, it was not sufficient alone, to increase in vivo tumorigenesis to male Cas9 control levels. The combination of p16 and p21 loss was sufficient to increase in vivo tumorigenesis without any additional increase in clonogenic cell frequency beyond that observed with p16 deletion. Thus, we concluded that cooperativity between these factors is necessary to both protect against aberrant proliferation, and the acquisition of new DNA mutations. Importantly, the induction of p16 and p21in female GBM astrocytes occurs in the absence of p53 function. Though not as potently, multiple other pathways can induce p21 expression in the absence of p53 function [1, 16, 17]. In addition, multiple other regulators of p53 function, such as MDMS and MDM4, are known to be altered in GBM and could also contribute to sex differences in p53 function and response to treatment.

While loss of p16, p21 or p27 equally abrogated sex differences in Rb phosphorylation, they did not have equivalent effects on in vivo tumorigenesis or in vitro clonogenic cell activity. In particular, p27 deletion significantly increased Rb phosphorylation without concomitant increases in clonogenic cell function or tumorigenesis. Combined loss of p16 and p21 was the only condition sufficient to render female GBM astrocytes like their male counterparts across all assays. This was clear in the in vivo tumorigenesis studies of GBM astrocytes rendered null for p16, p21 and p27 alone or in combination. The direct consequence of maintaining p16 and p21 function is the more normal response to the loss of growth factor signaling or the induction of DNA damage. The central importance of sexual dimorphism in cell cycle regulation and DNA repair was confirmed by the differences in male and female GBM astrocyte responses to etoposide treatment in which we observed sex differences in growth arrest, and substantial differences in the acquisition of chromosomal fragments in dividing cells.

Not only did etoposide treatment result in greater numbers of chromosomal aberrations, the fact that male cells did not undergo growth arrest, means that these aberrant chromosomal fragments are likely to be propagated. In light of new insights into the importance of extrachromosomal DNA to cancer evolution, this may be particularly important [42]. Finally, the sex differences in response to Palbociclib and etoposide strongly suggest that we must be thinking about incorporating the potential for sex differences into clinical trial design and interpretation.

In aggregate, the effect of p16 and p21 loss on the growth and tumorigenesis assays and transcriptomic analyses indicate that sex differences in cell cycle regulation and DNA repair may be central to sex differences in cancer incidence. Moreover, these new data speak broadly to the relatedness between growth regulation and cancer risk [2, 40]. Growth differences in male and female mammalian embryos are measurable from the moment of fertilization [3, 6, 8]. By the time of implantation, male human embryos contain greater numbers of cells than female embryos [19, 32, 41, 43]. In addition, male embryos consume more glucose and produce more lactate and pyruvate [11, 18, 39]. Sex differences in growth rates persist until adulthood resulting in statistically significant differences in normal adult male and female height. A difference in adult male and female size is present throughout primates and can be as large as 4:1.

Ultimately, the importance of studying sex differences in cancer will be determined by whether it improves outcomes. While outcome, including the quality of life and the total numbers of survivors, is likely to be improved by greater understanding of how sex differences impact on drug delivery, drug metabolism, toxicity and recovery, these data indicate that the potential for tumor cell intrinsic sex differences in drug sensitivity must also be accounted for in the plans and execution of clinical trials.

## Methods

### CRISPR-IUE Glioma model

Primary mouse glioma was generated using in utero electroporation (IUE) in the CD-1 IGS background as previously described [21]. Briefly, while under anesthesia, the uteri of timed pregnant females were exposed at embryonic stages E15–16. Two pX330 vectors [13] with guide sequence inserts targeting NF1 and p53 were injected (at a concentration of 1.5 μg/μl each) into the lateral ventricles, and progenitor cells were targeted via bioelectroporation (6, 55 msec pulses, at 33 V, set at 100 msec intervals). Additionally, electroporated cells were also labeled with the pGlast-PBase and PBCAG-GFP vectors for fluorescent visualization. Surgical incisions were sutured closed. Mice recovered and gave birth. Electroporated offspring were then monitored for abnormal behavioral symptoms (including but not limited to poor grooming, paralysis, hunched back, abnormal gate) or for megalencephaly/hydrocephaly, suggestive of tumor growth. Upon death, tumors were identified by their GFP fluorescence and diagnosed as glioblastoma by a neuro-pathologist using standard histological approaches.

### Immunohistochemistry and GFAP staining of mouse tumor tissues

Tissue samples were dissected and drop fixed in paraformaldehyde overnight. After embedding the tumor brains in a paraffin block, brains were sectioned and mounted. Histological features were analyzed through hematoxylin and eosin staining or immunohistochemistry against GFAP (1:1000; Dako, clone Z0334). Immunohistochemical signal was developed with the ImmPACT DAB Peroxidase Kit (Vector Laboratories, SK- 4105).

### RNA Seq library construction and sequencing

Total RNA was isolated using the RNeasy Mini Kit from Qiagen (Hilden, Germany) from GBM astrocytes treated with DMSO (0.05%) or Etoposide at 10 μg/ml for 4 h. PolyA Selection was performed to create RNA Seq libraries. mRNA was extracted from total RNA using a Dynal mRNA Direct kit. Sequencing was performed using HiSeq 1 × 50. RNA-seq reads were aligned to the Ensembl release 76 top-level assembly with STAR version 2.0.4b. Gene counts were derived from the number of uniquely aligned unambiguous reads by Subread:featureCount version 1.4.5. Transcript counts were produced by Sailfish version 0.6.3. Sequencing performance was assessed for total number of aligned reads, total number of uniquely aligned reads, genes and transcripts detected, ribosomal fraction known junction saturation and read distribution over known gene models with RSeQC version 2.3.

All gene-level and transcript counts were then imported into the R/Bioconductor package EdgeR and TMM normalization size factors were calculated to adjust for samples for differences in library size. Genes or transcripts not expressed in any sample were excluded from further analysis. The TMM size factors and the matrix of counts were then imported into R/Bioconductor package Limma and weighted likelihoods based on the observed mean-variance relationship of every gene/transcript and sample were then calculated for all samples with the voom With Quality Weights function. Performance of the samples was assessed with a spearman correlation matrix and multi-dimensional scaling plots. Gene/transcript performance was assessed with plots of residual standard deviation of every gene to their average log-count with a robustly fitted trend line of the residuals. Generalized linear models were then created to test for gene/transcript level differential expression. Differentially expressed genes and transcripts were then filtered for FDR adjusted *p*-values less than or equal to 0.05.

### Heat maps and pathway analysis

Heat map analysis was performed using GENE-E software (Broad Institute). Pathway enrichment analysis for differentially regulated genes was performed using a combination of KEGG pathway and Genomatix Pathway System (GePS). GePS uses information extracted from public and proprietary databases to display canonical pathways and to create and extend networks based on literature data. These sources include NCI-Nature Pathway Interaction Database, Biocarta, Reactome, Cancer Cell Map, and the ENCODE Transcription Factor project data. All data for pathway analysis is presented with adjusted corrected *p-*values.

### TCGA gene expression data analysis

To investigate whether the concordance of expression in mouse and human GBM samples could be due to random chance, we randomly selected 500 differentially expressed mouse genes and calculated the chance of having the same expression pattern in human TCGA samples. We then performed this random selection 100,000 times, fitted the data to normal distribution in R program and calculated the probability of having 50% concordance to be less than 10^− 6^.

### Growth assays, serum deprivation, Etoposide treatment

Growth kinetics of male and female GBM astrocytes with various genetic manipulations were examined by counting live cell number using an automated T4 cell counter as previously described with minor modifications [37]. Briefly, cells were harvested and plated in a 6-well plate at a density of 2 × 10^4^ cells/well (duplicates/treatment/genotype/time point/per each independent experiment) in 10% FBS supplemented DMEM/F12 media. 4-h post plating, cells were harvested by trypsinization and counted in the presence of trypan blue. This time point was designated as the starting point (T0) of the time course. Cells were then harvested and counted every 24 h for a total of 4 days (24, 48, 72 and 96 h). For serum deprivation experiments, 10% FBS supplemented media was replaced with DMEM/F12 media containing no serum or 0.1% FBS as indicated. For etoposide treatment, GBM astrocytes were treated with 10 μg/ml etoposide for 24 h, at which time media was changed to DMEM/F12 with 10% FBS.

### Quantitative real-time PCR

Total RNA was isolated using Trizol RNA extraction method (Invitrogen, CA). cDNA was generated using the SuperScript III reverse transcriptase (Invitrogen) and a mixture of poly-dT and random hexamer primers. Quantitative RT-PCR was performed using gene-specific primers (Additional file 1: Table S1) and iTaq SYBR Green PCR master mix (Biorad, CA). Data was analyzed by standard ΔCq method (2^-ΔΔCq^) where ΔCq is the difference between the gene of interest and GAPDH control Cq value. Similar experimental design was used for GBM astrocytes grown in the presence of serum or serum starved for 48 h.

### Western blot analysis

Rb phosphorylation kinetics was measured over 24 h of serum recovery after 48 h of serum starvation as previously described [37] in male and female *Nf1−/-DNp53* cells including Cas9 control and all KO lines. Total cell lysates (40 μg/lane) from each time point were separated by electrophoresis on 4–12% gradient Tris-Bis NuPAGE gels (Invitrogen) and transferred to a nitrocellulose membrane for immuno-blotting. After the membrane was blocked using blocking buffer (Odyssey blocking buffer 1:1 diluted in 0.1% PBST), primary antibodies diluted in the blocking buffer were incubated overnight at 4 °C. Secondary antibodies were diluted in blocking buffer and added to the membrane for 1 h at room temperature (in the dark). All blots were scanned by the Odyssey Infra-Red imaging system (LI-COR) and data were analyzed using the Odyssey software v3.0. Similar experimental design was used for GBM astrocytes treated with DMSO (0.05%) or Etoposide at 10 μg/ml for 24 h. Dilution factors for both primary and secondary antibodies are included in the Additional file 1: Table S2.

### EDU incorporation flow Cytometry

After incubation with 10 μM EdU, cells were fixed in ethanol and resuspended in PBS containing 1% FBS and stored at 4 °C. 3 × 10^5^ cells were strained through a 70-μm filter, and the manufacturer's protocol for Click-It EdU Plus Kit (Invitrogen) was followed. In brief, cells were permeabilized in PBS containing 0.1% Saponin and 1% BSA. Following permeabilization, 100 μL of Click-It Plus reaction mix was added, and the reaction was allowed to proceed for 30 mins. Cells were washed in PBS with 1% BSA and resuspended in PBS containing 0.015 mg/mL Propidium Iodide (PI, Invitrogen) and 0.025 mg/mL RNase A (Invitrogen) for 1 h. All samples were analyzed on the BD Fortessa using BD FACSDiva Software. To ensure consistency, a standard was run with each experiment.

To determine EdU incorporation and DNA ploidy, data was analyzed using FlowJo. The cell gating paradigm was consistent across experiments. In brief, cells were first gated on FSC-A vs SSC-A followed by gating on FSC-A vs FSC-W to remove doublets. While most cells were diploid or tetraploid, higher ploidy cells were inconsistently evident on the PI gate (PE channel) and a box was drawn to remove these from the analysis.

### Chromosome analysis: Karyotyping

Chromosome analysis was performed on male and female GBM astrocytes treated with DMSO (0.05%) or Etoposide at 10 μg/ml for 4 h. Cells were allowed to recover for 24–48 h in serum supplemented media following treatment to ensure active division. At that time, cells were treated in hypotonic solution, fixed and stained for GTG banding and microscopic evaluation per standard procedures in the Cytogentics Core laboratory of Washington University School of Medicine. Twenty metaphases for each cell and condition were counted in order to enumerate the range of chromosomes. Three metaphase spreads from each cell line and condition were digitally processed for detailed karyotyping.

### Cells, constructs and CRISPR design

Male and female GBM (*Nf−/−;DNp53*) astrocytes were generated as previously reported [37] and cells were grown in DMEM/F12 media supplemented with 10% FBS and 1% penicillin-streptomycin. CRISPR/Cas9 based gene depletion was achieved in the GBM astrocytes using LentiCRISPRv2 (Addgene plasmid: 52,961 by Dr. Feng Zhang) derived lentiviruses. To express p27-specific guide RNA, we replaced the Cas9 coding region in the LentiCRISPRv2 backbone with mCherry as a flow-sorting marker. We cloned the guide RNA (sgRNA) targeting the gene of interest into the gRNA expression system as described [30]. All sgRNAs were designed using the publicly available CRISPR design tool (http://www.broadinstitute.org/rnai/public/analysis-tools/sgrna-design). The top three sgRNAs with the highest scores and the least off-target potential were selected for further functional screening. Guide RNA sequences are listed in Additional file 1: Table S1.

### Knockout (KO) of p21, p27 and p16 in GBM Astrocytes

p21 knockout (p21-KO) lines were generated in the *Nf1−/−;DNp53* astrocytes by infecting them with the lentiviral Cas9/sgRNA-p21 all-in-one construct. *Nf−/−;DNp53-Cas9* control lines were made by viral infection with lentiviral Cas9 vector without any guide RNA. Both the Cas9 control and p21-KO lines were selected by puromycin (2.5 μg/ml) media for 1 to 2 weeks and surviving cells were expanded for target knockout analysis. To knockout p27, we first made a p27-specific sgRNA lentiviral vector by replacing Cas9 gene with an mCherry coding sequence. Then, we lentivirally expressed the p27-specific sgRNA in the *Nf1−/−;DNp53*-Cas9 control lines and mCherry positive cells were sorted by flow-cytometry (Synergy, SONY Biotechnology). Similar methodology was used to prepare the *p16* KO lines. To generate the double and triple knockout lines (*p21;p27-DKO*, *p21;p16-DKO*; *p27;p16 -DKO* and *p21;p27;p16 triple KO*), *p21-KO* lines were infected with p27 or p16-specific sgRNA lentiviral vector and selected for mCherry expression. *p21;p27;p16 triple KO* lines were generated by infecting the *p21;p27 DKO* lines with p16-specific sgRNA lentiviral vector and passaging for at least 5 passages for selection purposes. Gene knockout efficiency was assessed by Western blot after the knockout lines were passaged three times, and cells with > 80% knockout of target protein were used for the functional assays.

### In vivo tumorigenesis: Flank implantations

Flank tumors were generated by implanting GBM astrocytes with each of the genetic manipulations subcutaneously into left and right-side flanks. These cells were treated with EGF for 1 week (50 ng/ml). One million cells were then harvested and resuspended in 100 μl of 1:1 media to matrigel (BD Biosciences) and injected into the two flanks of mice. In some cases, a third flank xenograft of male GBM cells was included as a positive control. Equal numbers of mice received either Cas9 control or KO cells. We have previously shown that male cells but not female cells grow tumors in vivo, irrespective of the sex of the recipient mice, thus only female mice were used in this study [37].

### Clonogenic cell frequency assay: Extreme limiting dilution assays (ELDA analysis)

Clonogenic capacity of male and female GBM astrocytes with various genetic manipulations was assayed by the Extreme Limiting Dilution Assay (ELDA). The frequency of clonogenic stem cells was evaluated by the cells’ ability to form tumor-spheres in low-adherent conditions as previously reported [37]. Briefly, cells were harvested into a single cell suspension and plated in neurosphere media containing EGF and FGF on 96-well ultra-low attachment plates in a serial dilution ranging from 3000 cells/well to 1 cell/well (3000, 600, 120, 24, 5 and 1 cells; *n* = 14/cell density). Sphere formation was measured 7 days after plating. Clonogenic stem-like cell frequency was analyzed using the Extreme Limiting Dilution Analysis software (http://bioinf.wehi.edu.au/software/elda/).

### Statistical analysis

All experiments in this study were carried out at least three times using three independent preparations of *Nf1−/−;DNp53* astrocytes. Two-way ANOVA was used to evaluate differences in cell growth over time. One-way ANOVA with a post-hoc Dunnett’s correction was used to compare the differences in all functional measurements between p21, p27 and/or p16 knockout vs. control group. Survival data were evaluated with log-rank test. In all cases a *p*-value < 0.05 was considered statistically significant.

### Study approval

Animals were used in accordance with an animal studies protocol (no. 20150177) approved by the Animal Studies Committee of the Washington University School of Medicine per the recommendations of the Guide for the Care and Use of Laboratory Animals (NIH).

## Additional file

Additional file 1: **Table S1.** Real-time quantitative PCR primers and CRISPR guide RNA sequences. **Table S2.** Antibody details. **Figure S1.** Female and male CRISPR-IUE gliomas exhibit differences in histology. **Figure S2.** Sex differences in p21 and p27 expression in the CRISPR-IUE glioma model. **Figure S3.** Metaphase spreads from male and female GBM astrocytes grown under control (DMSO treated) conditions revealed aneuploidy. **Figure S4.** Western blot demonstration of p16, p21 and p27 deletion in Cas-9 expressing male and female GBM astrocytes expressing (+) or not expressing (−) the appropriate guide RNAs (gRNAs) as indicated. (PDF 3121 kb)
