# Supplementary material for: Cooperative p16 and p21 action protects female astrocytes from transformation
Source: Acta Neuropathol Commun. 2018 Feb 20;6:12. doi: 10.1186/s40478-018-0513-5 (PMC5819173; doi:10.1186/s40478-018-0513-5)
Supplement: Supplementary file 1 — Table S1. Real-time quantitative PCR primers and CRISPR guide RNA sequences. Table S2. Antibody details. Figure S1. Female and male CRISPR-IUE gliomas exhibit differences in histology. Figure S2. Sex differences in p21 and p27 expression in the CRISPR-IUE glioma model. Figure S3. Metaphase spreads from male and female GBM astrocytes grown under control (DMSO treated) conditions revealed aneuploidy. Figure S4. Western blot demonstration of p16, p21 and p27 deletion in Cas-9 expressing male and female GBM astrocytes expressing (+) or not expressing (−) the appropriate guide RNAs (gRNAs) as indicated. (PDF 3121 kb) [file 40478_2018_513_MOESM1_ESM.pdf]

Kfoury, Sun *et al.* Supplemental Material

Contents:

Supplemental Table 1
Supplemental Table 2

Supplemental Figure 1
Supplemental Figure 2
Supplemental Figure 3
Supplemental Figure 4

Supplemental Table 1: Real-time quantitative PCR primers and CRISPR guide RNA sequences			
**Target**	Forward (qPCR)	Reverse (qPCR)	CRISPR gRNA (20nt)
**Cdkn1A (p21)**	AGACATTCAGAGCCACAGGCACCA	GCATCGCAATCACGGCGCAA	ACTTCGTCTGGGAGCGCGTT
**Cdkn1B(p27)**	TGGTGGACCAAATGCCTGAC	TTCGGGGAACCGTCTGAAAC	GGACTTGGAGAAGCACTGCC
**Cdkn2A (p16)**	GACATCGTGCGATATTTGCGTTCCG	TTTAGCTCTGCTCTTGGGATTGGCC	GGTACACAAAGACCACCCAG
**GAPDH**	TGGAGTCTACTGGTGTCTTCACCACCAT	GTGGCAGTGATGGCATGGACTGTG	NA

Supplemental Table 2: Antibody details		
**Antibody**	Manufacturer	Dilution
**Rabbit anti pan-Rb**	Cell Signaling Technology	1:1000
**Rabbit anti phospho-Rb (Ser807/811)**	Cell Signaling Technology	1:200
**Rabbit anti-p21**	Santa Cruz	1:300
**Rabbit anti-p27**	Cell Signaling Technology	1:1000
**Mouse anti-beta Actin**	Sigma	1:50,000
**Mouse anti-p16**	Santa Cruz	1:200
**Donkey anti-rabbit IRdye800 (secondary)**	Li-Cor	1:50,000
**Donkey anti-mouse IRdye680 (secondary)**	Li-Cor	1:50,000

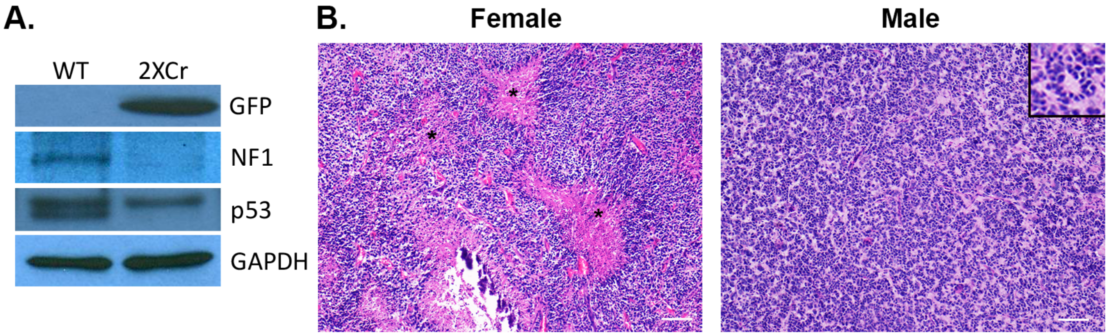

Supplemental Figure 1: Female and male CRISPR-IUE gliomas exhibit differences in histology. (**A**) Deletion of *Nf1* and *p53* in CRISPR-IUE tumors was confirmed by Western blot analysis. GFP provides confirmation that the analysis is of transduced cells. GAPDH provides loading control. (**B**) Female tumors exhibited greater degrees of necrosis (asterisks) while male tumors exhibited greater numbers of tumor cell rosettes (inset), a primitive neuro-ectodermal feature. Scale bars = 100 μM.

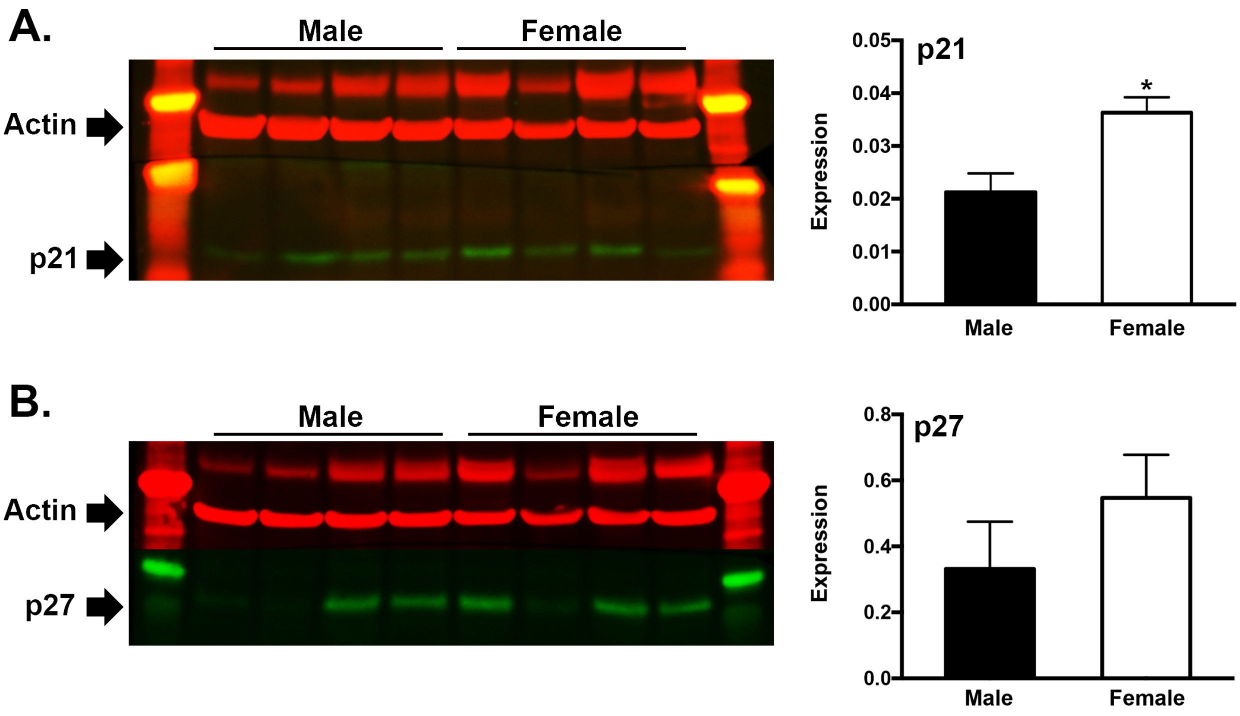

Supplemental Figure 2: Sex differences in p21 and p27 expression in the CRISPR-IUE glioma model. Tumors were recovered from male (n=4) and female (n=4) mice at the time of euthanasia. Protein lysates were prepared and Western blot analysis for actin (loading control), p21 (**A**) and p27 (**B**) were performed by standard procedures. Left-hand panels are blots and right-hand panels are quantification of mean and SEM of the ratios of p21/actin or p27/actin. * = $p < 0.05$ as determined by two-tailed *t*-test.

DMSO Treated

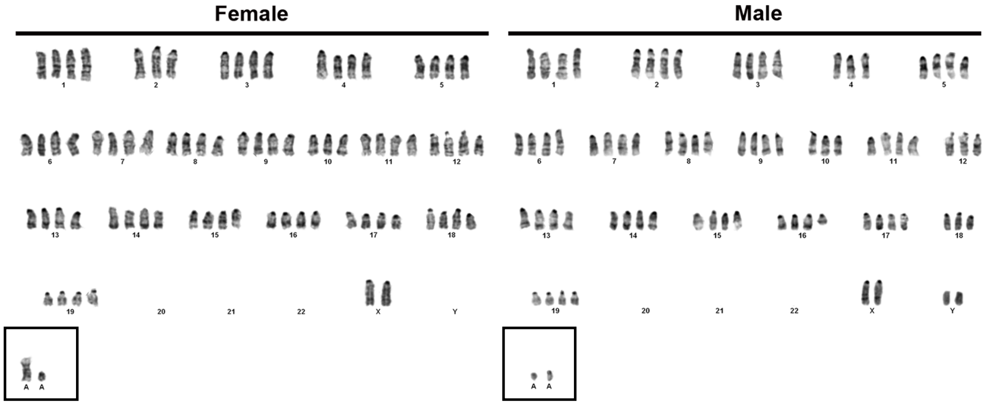

Treatment	Sex and Litter Number	Range in Chromosome Number
DMSO		
	M6	50-105
	M7	52-83
	M8	54-120
	F6	41-109
	F7	44-99
	F8	58-111
Etoposide		
	M6	62-105
	M7	60-82
	M8	72-80
	F6	39-81
	F7	60-153
	F8	76-82

Litter number refers to the litter from which the astrocytes were derived. All analyses involved astrocytes derived from three independent litters of mice.

Supplemental Figure 3: Metaphase spreads from male and female GBM astrocytes grown under control (DMSO treated) conditions revealed aneuploidy. Range in chromosome numbers for both the DMSO- and Etoposide-treated conditions are shown in the table. M refers to male and F to female astrocytes. Numbers 6-8 refer to the litters from which the astrocytes were derived. For both male and female GBM astrocytes, there were two identified chromosomal structural variants identified (boxed elements in spreads).

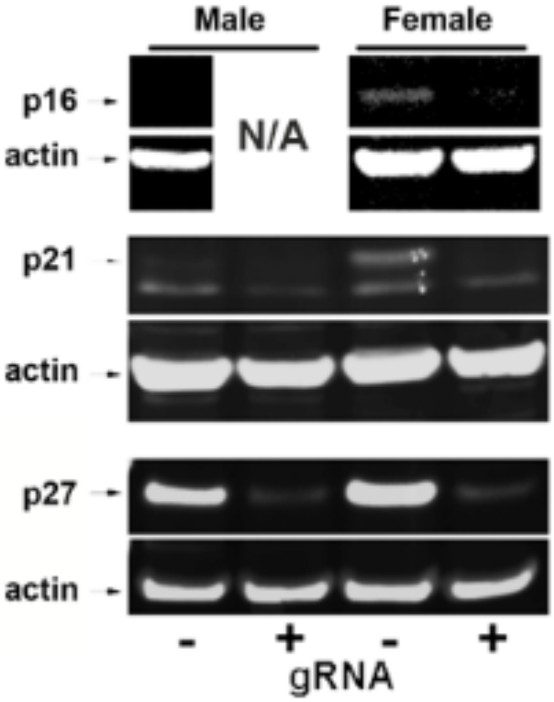

Supplemental Figure 4: Western blot demonstration of p16, p21 and p27 deletion in Cas-9 expressing male and female GBM astrocytes expressing (+) or not expressing (-) the appropriate guide RNAs (gRNAs) as indicated.
